# Electron transport and light-harvesting switches in cyanobacteria

**DOI:** 10.3389/fpls.2014.00007

**Published:** 2014-01-21

**Authors:** Conrad W. Mullineaux

**Affiliations:** School of Biological and Chemical Sciences, Queen Mary University of LondonLondon, UK

**Keywords:** cyanobacteria, electron transport, light-harvesting, orange carotenoid protein, phycobilisome, state transitions, thylakoid membrane

## Abstract

Cyanobacteria possess multiple mechanisms for regulating the pathways of photosynthetic and respiratory electron transport. Electron transport may be regulated indirectly by controlling the transfer of excitation energy from the light-harvesting complexes, or it may be more directly regulated by controlling the stoichiometry, localization, and interactions of photosynthetic and respiratory electron transport complexes. Regulation of the extent of linear vs. cyclic electron transport is particularly important for controlling the redox balance of the cell. This review discusses what is known of the regulatory mechanisms and the timescales on which they occur, with particular regard to the structural reorganization needed and the constraints imposed by the limited mobility of membrane-integral proteins in the crowded thylakoid membrane. Switching mechanisms requiring substantial movement of integral thylakoid membrane proteins occur on slower timescales than those that require the movement only of cytoplasmic or extrinsic membrane proteins. This difference is probably due to the restricted diffusion of membrane-integral proteins. Multiple switching mechanisms may be needed to regulate electron transport on different timescales.

## THE ENVIRONMENT IN AND AROUND CYANOBACTERIAL THYLAKOID MEMBRANES

The thylakoid membranes of cyanobacteria are a complex intracytoplasmic membrane system that houses the photosynthetic electron transport chain, and also most of the respiratory electron transport complexes. Thylakoid membrane pairs separate a lumenal space from the cytoplasm: electron transport results in proton translocation across the membrane and into the lumen, and the resultant proton motive force is used to power ATP synthesis. Structural studies by cryo-electron tomography suggest that there may be limited connections between the thylakoid and plasma membranes: the nature of these connections remains unclear (Liberton et al., 2006; van de Meene et al., 2006, 2012). Multiple thylakoid membrane layers can be connected by membrane bridges, suggesting that the thylakoid membrane forms a single continuous surface enclosing a single, interconnected lumen (Nevo et al., 2007). The topology of the membrane varies in different species, but generally it forms a series of lamellar membrane layers located between the cytoplasmic membrane and a central cytoplasm that houses the nucleoid and other bodies such as carboxysomes (Liberton et al., 2006).

Like other photosynthetic membranes (Kirchhoff et al., 2008), cyanobacterial thylakoid membranes are densely packed with membrane-integral proteins (Folea et al., 2008). The protein complexes present at the highest concentrations are the Photosystem I and Photosystem II reaction centers. Photosystem I is normally trimeric, while Photosystem II is dimeric and sometimes arranged in rows (Vernotte et al., 1990). Different membrane layers may have somewhat different proportions of Photosystem I and Photosystem II, with implications for the predominant modes of photosynthetic electron transport in those membrane regions (Sherman et al., 1994; Vermaas et al., 2008). In contrast to green plant chloroplast thylakoids, there are no stacked grana regions and no obvious lateral heterogeneity in the distribution of the photosystems. Most of the membrane area is devoted to photosynthetic electron transport and occupied by photosynthetic complexes, with Photosystem II and Photosystem I reaction centers often in close proximity (Folea et al., 2008). However, there are indications of discrete, functionally specialized regions of the membrane interspersed among the bulk membrane devoted to photosynthetic electron transport. For example, it is possible to isolate a membrane fraction of intermediate density devoted to reaction center biogenesis, perhaps located at the interface between the thylakoid and cytoplasmic membranes (Stengel et al., 2012). Fluorescent protein tagging of minor thylakoid membrane components, combined with fluorescence microscopy, sometimes reveals proteins clustered in distinct zones in the membrane, with very different distribution to the photosynthetic reaction centers. The respiratory electron transport complexes Complex I (Ndh-1) and succinate dehydrogenase (Sdh) are clustered in specific membrane zones under some conditions (Liu et al., 2012), as is the membrane-integral PSII repair protease FtsH2 (Komenda et al., 2006). These studies indicate a degree of lateral heterogeneity in the cyanobacterial thylakoid membrane, suggesting the presence of localized, functionally specialized islands.

The dense packing of proteins in cyanobacterial thylakoids restricts the mobility of membrane-integral proteins. Fluorescence recovery after photobleaching (FRAP) measurements show that Photosystem II centers are almost immobile under normal conditions (Mullineaux et al., 1997). Another chlorophyll-binding protein, IsiA, can diffuse more freely than Photosystem II, but its diffusion coefficient is still low compared to *Escherichia coli *plasma membrane proteins, for example (Sarcina and Mullineaux, 2004). Restricted protein mobility has implications for any processes involving protein redistribution, including the biogenesis, turnover, and repair of the photosynthetic reaction centers, and regulation of electron transport and light-harvesting (Mullineaux, 2008a). In contrast to the reaction centers, the phycobilisome light-harvesting complexes on the cytoplasmic surface of the membrane are rather freely mobile, despite their large size (Mullineaux et al., 1997; Sarcina et al., 2001). Therefore the environment in the aqueous phase on the cytoplasmic side of the thylakoid membrane is rather fluid, potentially allowing large cytoplasmic proteins to visit the membrane surface quite freely. On the other side of the membrane, we have little information on the fluidity of the thylakoid lumen in cyanobacteria, although this question is of considerable interest for understanding electron transport mediated by lumenal electron carriers (Kirchhoff et al., 2011). The electron tomographic study of Nevo et al. (2007) suggests the lumen is highly connected.

## REGULATORY SWITCHES IN CYANOBACTERIAL PHOTOSYNTHESIS

**FIGURE 1:**
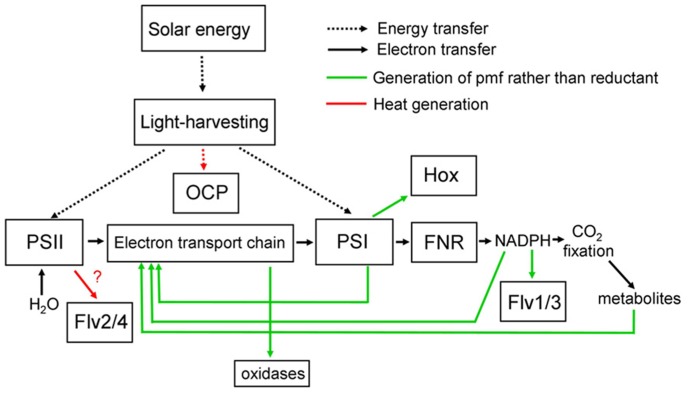
** Simplified scheme for light-harvesting and electron transport in cyanobacteria, showing alternative pathways and their principle functional consequences**. Note that the functional consequences of electron transfer to Flv2/4 are uncertain, as the subsequent electron acceptor is unknown (Zhang et al., 2012). See text for further discussion and references. Flv, Flavodiiron protein; FNR, ferredoxin-NADP oxidoreductase; Hox, bidirectional [NiFe] hydrogenase; OCP, orange carotenoid protein; pmf, proton-motive force; PSI, Photosystem I; PSII, Photosystem II.

**FIGURE 2:**
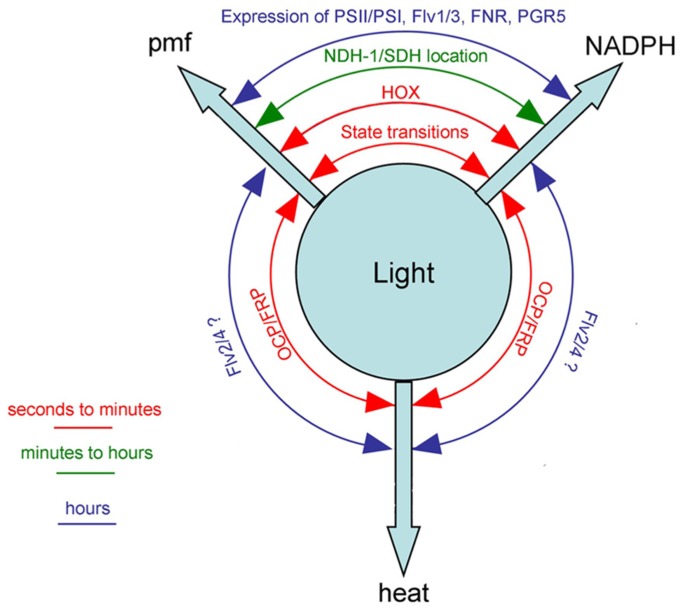
** Cyanobacterial mechanisms for switching between the three principal outputs of photosynthesis [proton-motive force (pmf), reducing power (NADPH) and heat] with the approximate timescales on which they occur**. See text for discussion and references. FRP, fluorescence recovery protein; NDH-1, complex I/NAD(P)H dehydrogenase; SDH, complex II/succinate dehydrogenase. Other abbreviations defined in the legend to **Figure 1**.

Light energy absorbed by the photosynthetic pigments in the thylakoid membranes can be used for the generation of reduced NADPH with electrons extracted from water by Photosystem II. NADPH then supplies reducing power for anabolic processes such as the Calvin–Benson cycle for CO_2_ fixation. Additionally, energy is stored in the form of a proton gradient across the thylakoid membrane, with the proton-motive force used to power the synthesis of ATP from ADP and phosphate. The ATP is required for energy input into the Calvin–Benson cycle, but can also be employed in many other cell processes. Absorbed light energy can also be dissipated as heat, via a variety of direct and indirect routes. **Figure 1** summarizes, in highly simplified form, some of the alternative pathways of energy conversion in and around cyanobacterial thylakoid membranes, with their outputs of reducing power, proton-motive force or heat. The balance of photosynthetic outputs is critically important for the physiology of the cell. In particular, the cell has to maintain an appropriate redox balance, with sufficient reducing power available for CO_2_ fixation and other anabolic reactions, but without over-reduction of the electron transport chain, which can lead to dangerous side-reactions with oxygen (Murata et al., 2012). Cyanobacteria possess a remarkable number of diverse mechanisms to adjust the balance of photosynthetic outputs, which are discussed in more detail below. The switches work either through regulation of photosynthetic light-harvesting (discussed in Section “Regulation of Light-harvesting”) or through direct regulation of electron transport pathways (discussed in Section “Regulation of Electron Transport”). **Figure 2** summarizes some known regulatory switches, their effects on the balance of photosynthetic outputs, and the timescales on which they operate.

## REGULATION OF LIGHT-HARVESTING

In most cyanobacteria, the principal light-harvesting complexes are phycobilisomes, large protein assemblies anchored to the cytoplasmic surface of the thylakoid (Watanabe and Ikeuchi, 2013). Phycobilisomes contain phycobiliproteins with chromophores which may absorb light in the range from about 500 to 670 nm. Energy absorbed by the phycobilisomes can be directed to either Photosystem II or Photosystem I reaction centers (Mullineaux, 2008b; Liu et al., 2013). State transitions are a mechanism that controls the relative extent of energy transfer from phycobilisomes to Photosystem I vs. Photosystem II, by a process likely involving the physical movement of phycobilisomes on the membrane surface (Joshua and Mullineaux, 2004) and possibly also involving changes in the “spillover” of energy from Photosystem II to Photosystem I (McConnell et al., 2002). They are a rapid, post-translational mechanism acting on a timescale of seconds to a few minutes. The signal transduction pathway is not established, but it is clear that state transitions are triggered by a redox signal: reduction of plastoquinone triggers adaptation to “state 2” in which more energy is transferred to Photosystem I. Oxidation of plastoquinone triggers the opposite effect, with more energy transferred to Photosystem II (Mullineaux and Allen, 1990). Cyanobacteria exhibit several modes of cyclic electron transport around Photosystem I, in which electrons are returned by various routes from the acceptor side of Photosystem I back to the donor side of Photosystem I via the photosynthetic electron transport chain (Yeremenko et al., 2005; Battchikova et al., 2011). These pathways do not involve Photosystem II, and therefore do not result in the generation of reducing power by extraction of electrons from water. Instead they result only in the generation of a proton-motive force. By changing the balance of energy transfer to the photosystems, state transitions act to control the balance of linear electron transport (involving both photosystems and generating both reducing power and a proton-motive force) and Photosystem I-cyclic electron transport (involving Photosystem I only and generating only proton-motive force). The transition to State 2 switches photosynthetic output to increased production of proton-motive force at the expense of reducing power, while the transition to State 1 will have the opposite effect (**Figure 2**). The redox trigger for state transitions allows the switch to be controlled by the redox status of the cell.

A second light-harvesting switch in cyanobacteria involves the orange carotenoid protein (OCP), a photoactive cytoplasmic protein whose conformation is switched by exposure to blue light (reviewed by Kirilovsky and Kerfeld, 2013). Because the light-induced conformation is unstable, a high proportion of the photoactivated state can only be maintained by strong illumination and therefore the OCP acts as a high-light sensor. The photoactivated OCP binds to the core of the phycobilisome, where it takes excitation energy from the phycobilins and converts it to heat. Therefore it acts as a simple but highly effective light-activated energy quencher, preventing photodamage of the reaction centers at high light intensities. OCP diverts energy away from both photosystems, and serves to switch photosynthetic outputs to heat at the expense of generation of both proton-motive force and reducing power (**Figure 2**). The reversal of OCP-induced energy quenching depends on a second cytoplasmic protein, the fluorescence recovery protein (FRP), which binds to the OCP and weakens its association with the phycobilisomes (Kirilovsky and Kerfeld, 2013). OCP energy quenching is rapidly induced and reversed, on a timescale of seconds to minutes. In contrast to state transitions, there appears to be no feedback between the metabolic status of the cell and the OCP light-harvesting switch: the switch occurs simply in response to light intensity.

## REGULATION OF ELECTRON TRANSPORT

Light-harvesting switches (Section “Regulation of Light-harvesting”) have obvious, but indirect, effects on photosynthetic electron transport. Another set of switches in cyanobacteria directly influence the pathways of electron transport by controlling the stoichiometry, interactions or proximity of different electron transport components in and around the thylakoid membrane. These switches can act to control the balance of linear and cyclic electron transport, and therefore the balance of proton-motive force and reducing power as photosynthetic outputs. Electron transport switches can also serve to generate heat at the expense of other photosynthetic products. For example, diverting electrons from the photosynthetic electron transport chain back to oxygen as an electron acceptor will generate proton-motive force and/or heat at the expense of reducing power. Switches that remove electrons from the photosynthetic electron transport chain are also known as “electron valves”: they apparently serve to prevent dangerous over-reduction of the electron transport chain. The flavodiiron proteins Flv1–4 are examples of cyanobacterial electron valves: they are cytoplasmic proteins that take electrons from the photosynthetic electron transport chain and divert them to alternative acceptors. Flv1 and Flv3 form a heterodimer that takes electrons from the acceptor side of PSI and uses them to reduce oxygen (Allahverdiyeva et al., 2013). An Flv2/Flv4 heterodimer takes electrons from the acceptor side of Photosystem II, passing them to an unknown acceptor (Zhang et al., 2012). The activities of the Flv proteins are not known to be regulated, except through their expression level. Therefore they presumably act only as an electron transport switch on slow timescales (**Figure 2**). Another cyanobacterial electron valve is the bidirectional hydrogenase (Hox), which can take electrons from the acceptor side of Photosystem I and use them to reduce protons to produce hydrogen (Appel et al., 2000; Gutekunst et al., 2013). Diffusion of hydrogen out of the cell will result in loss of reducing power. With both Hox and Flv1/3, consumption of protons in the cytoplasm must generate extra proton-motive force. Therefore these complexes serve as electron valves and also as switches from the generation of reducing power to the generation of proton motive force (**Figure 2**). Hox is inhibited by oxygen but active under anaerobic conditions: it may be important primarily as an anaerobic alternative to Flv1/3, able to remove electrons from the system when oxygen is unavailable to act as a sink for electrons from Flv1/3. Unlike the Flv proteins, Hox activity is very variable on short timescales, suggesting that it could be acting as a rapid electron-transport switch. Hox activity is observed as a short burst of hydrogen evolution when dark-adapted anaerobic cells are illuminated (Appel et al., 2000). Hydrogen evolution then ceases in parallel with the rise in oxygen concentration due to Photosystem II activity (Cournac et al., 2004). It remains to be determined whether this loss of activity simply results from direct oxygen inhibition of Hox, or whether it also reflects a regulatory mechanism controlling Hox activity. Hox is not irreversibly inactivated by O_2_ (McIntosh et al., 2011), suggesting that regulation is a possibility. Under aerobic conditions, the thylakoid terminal oxidases cytochrome *bd*-1 and cytochrome oxidase also appear to be important as electron valves, since null mutants are disadvantaged under fluctuating light (Lea-Smith et al., 2013).

A second area where there is potential for electron transport switching is in the control of cyclic vs. linear electron transport, which is critical for the redox balance of the cell (Section “Regulatory Switches in Cyanobacterial Photosynthesis”). Cyanobacteria have multiple routes for cyclic electron transport around Photosystem I (reviewed in Mullineaux, 2014). One route involves electron transfer from ferredoxin to plastoquinone via the cytoplasmic PGR5 protein (Yeremenko et al., 2005), while another likely route involves electron transfer from ferredoxin to plastoquinone via Ndh-1 and requiring the NdhS subunit (Battchikova et al., 2011). A route via ferredoxin-NADP oxidoreductase (FNR), NADPH, and Ndh-1 is also plausible. Control over the extent of cyclic vs. linear electron transfer could be exerted through the control over the expression of PGR5, NDH-1, and short and long isoforms of FNR, which have different localization, with possible effects on electron transport routes (Thomas et al., 2006). The newly identified NdhP subunit of Complex I is another factor whose expression could control pathways of cyclic vs. linear electron flow (Schwarz et al., 2013).

On shorter timescales, there is scope for faster post-translational mechanisms to switch between cyclic and linear electron transport. One such mechanism may control the cyclic electron transport pathway involving Complex I (Liu et al., 2012). Fluorescence microscopy combined with GFP-tagging of Complex I in the cyanobacterium *Synechococcus *sp. PCC7942 shows that the larger-scale distribution of the complex in the membrane is controlled in response to a redox switch. Oxidation of the plastoquinone pool induces the clustering of Complex I in segregated thylakoid membrane zones, while reduction of the plastoquinone pool induces a post-translational switch in the distribution of Complex I to a state in which it is more evenly dispersed in the membrane. Complex II (succinate dehydrogenase) shows a similar change in distribution under the same conditions. This switch in the distribution of the complexes correlates with a major change of the probability that electrons from the respiratory complexes are transferred to a Photosystem I rather than to a terminal oxidase (Liu et al., 2012). This provides a mechanism to promote cyclic electron transport when the reduction of the plastoquinone pool indicates an adequate supply of electrons in the cell. Although many questions remain about the mechanism, the observation indicates that the distribution of electron transport complexes in the membrane at the sub-micron scale is under physiological control, and plays a crucial role in controlling pathways of electron flow.

## MEMBRANE RE-ORGANIZATION AND FUNCTIONAL SWITCHES ON DIFFERENT TIMESCALES

The switching mechanisms discussed above all involve the physical relocation of protein components in or near the thylakoid membrane. Almost certainly, other switches remain to be characterized. However, from the switching mechanisms already characterized it is apparent that switches operate on different timescales, according to the kind of structural reorganization required.

The fastest switching mechanisms operate on timescales of seconds to a few minutes. These mechanisms are post-translational, and involve the movement only of cytoplasmic or membrane-extrinsic proteins. One example is OCP-mediated phycobilisome energy quenching, which requires OCP molecules to be photoconverted and then to bind to phycobilisomes on the membrane surface (Kirilovsky and Kerfeld, 2013). A second example is state transitions, which appear to operate largely through the movement of the membrane-extrinsic phycobilisomes, although limited rearrangements of membrane proteins may also be involved (Vernotte et al., 1990; Joshua and Mullineaux, 2004).

Post-translational switching mechanisms requiring substantial relocation of membrane-integral proteins occur on slower timescales. One example is the redistribution of Ndh-1 (see Section “Regulatory Switches in Cyanobacterial Photosynthesis”). This is triggered by a change in the redox state of plastoquinone, similarly to state transitions. However, the redistribution, and associated changes in electron transport, occur on a slower timescale of 10–30 min (Liu et al., 2012), as compared to about 1–5 min for state transitions (Joshua and Mullineaux, 2004). Since the trigger signal is the same, the slower timescale for Ndh-1 redistribution likely reflects the slow diffusion of membrane-integral proteins in the crowded thylakoid membrane. Another example is a redistribution of Photosystem II following exposure to intense red light, which requires around 8 min, likely due to the restricted diffusion of Photosystem II even after mobilization has been triggered (Sarcina et al., 2006).

The slowest switching mechanisms involve changes in gene expression. For example, changes in the cellular content of Photosystem I occur in response to a redox trigger (Fujita et al., 1988) similarly to state transitions (Mullineaux and Allen, 1990) and redistribution of Ndh-1 (Liu et al., 2012), however the timescale is substantially slower than the other effects, requiring about 20 h for completion (Fujita et al., 1988). Changes in the cellular content of GFP-tagged Ndh-1 can be similarly triggered and directly visualized by quantifying GFP fluorescence: more than 1 h is required for completion (Liu et al., 2012).

**Figure 2** summarizes some known photosynthetic switches in cyanobacteria, and the timescales on which they occur. It is intriguing that cyanobacteria have so many mechanisms to control photosynthetic electron transport, including multiple redox-triggered mechanisms to control the production of reducing power vs. proton motive force (**Figure 2**). The reason for this multiplicity of mechanisms may lie in the different timescales on which the different switches operate. Cyanobacteria can be exposed to changes in their light environment on multiple timescales, therefore need a suite of acclimation mechanisms ranging from rapid emergency responses to more optimal long-term changes in membrane protein content. Restricted protein mobility in the thylakoid membrane means that the fastest responses are always likely to involve membrane-extrinsic proteins.

## Conflict of Interest Statement

The author declares that the research was conducted in the absence of any commercial or financial relationships that could be construed as a potential conflict of interest.
